# Evaluation of the BD GeneOhm MRSA and VanR Assays as a Rapid Screening Tool for Detection of Methicillin-Resistant *Staphylococcus aureus* and Vancomycin-Resistant Enterococci in a Tertiary Hospital in Saudi Arabia

**DOI:** 10.1155/2011/861514

**Published:** 2011-12-01

**Authors:** H. Hassan, M. Shorman

**Affiliations:** ^1^Department of Pathology and Laboratory Medicine, King Fahad Specialist Hospital-Dammam (KFSHD), Dammam 31444, Saudi Arabia; ^2^Department of Microbiology, Alexandria University, Alexandria 2111, Egypt; ^3^Infectious Diseases section, King Fahad Specialist Hospital-Dammam (KFSHD), Dammam 31444, Saudi Arabia

## Abstract

*Objectives*. The aim of this study was to evaluate the diagnostic performance of BD GeneOhm VanR Assay, a rapid PCR test that detects the presence of *vanA* and/or *vanB* genes and the performance of BDGeneOhm MRSA Assay which detects the staphylococcal cassette chromosomemec (*SCCmec* cassette) carrying the *mecA* gene and *Staphylococcus aureus* specific sequence located within the *orfX* gene. *Methods*. 300 duplicate rectal swabs collected consecutively were analyzed for the presence of VRE by culture and BD PCR. 2267 duplicate swabs were collected (728 nasal and 1539 groin swabs) and analyzed for the presence of MRSA by culture method and BD PCR. *Results*. Compared to culture, the BD GeneOhm VanR Assay showed a sensitivity, specificity, positive predictive value (PPV) and negative predictive value (NPV) of 100%, 91.1%, 23.5%, and 100%, respectively. The BD GeneOhm MRSA Assay revealed sensitivity, specificity, PPV, and NPV of 97.2%, 99.4%, 89.7%, and 99.9%, respectively, for nasal swabs. For groin swabs, it was 100%, 98.7%, 61.5% and 100%, respectively. *Conclusion*. The BD GeneOhm vanR Assay is a good screening test for rapid exclusion of VRE carriers in hospitals. The BD GeneOhm MRSA Assay represents a reliable screening test. The true strength of the BD GeneOhm Assay for MRSA and VRE is its exceptionally high NPV making the test an ideal tool for rapid exclusion of MRSA and VRE carriers in hospitals. As a consequence, this would dramatically shorten the patient isolation time.

## 1. Introduction

Methicillin-resistant *staphylococcus aureus *(MRSA) and vancomycin-resistant enterococcus (VRE) are multidrug resistant organisms and are particularly frequent causes of hospital-acquired infections that are often difficult and expensive to treat [1]. 

Methicillin resistance in *S. aureus* is primarily mediated by the *mecA *gene, which codes for the modified penicillin-binding protein 2a (PBP 2a) [1]. Several studies on *S. aureus* suggest that MRSA infection usually follows prior carriage rather than occurring from direct transmission during invasive procedures by staff or from intensive care unit (ICU) environment [2–5], meaning that MRSA infection is preceded by colonization with an MRSA strain that is genetically indistinguishable from the disease causing isolation in at least 56% of patients. These data support the view that prevention of colonization of ICU patients with MRSA could reduce the frequency of MRSA infections and can assist in the design of effective prevention strategies against MRSA infection [6]. 

Enterococci inhabit the gastrointestinal tract and are considered normal flora. Their emergence as important pathogens in the past two decades is largely due to their resistance to many commonly used antibiotics [7]. Most enterococcal infections have been attributed to endogenous sources (normal flora of individual patient), yet transmission occurs by contaminated hands of personnel, contaminated patient-care equipment, and/or environmental surfaces. Treatment of some enterococcal infection has become a significant challenge, especially with the emergence of strains resistant to vancomycin [1]. A few genes are responsible for vancomycin resistance (intrinsic or acquired). The *vanA *and *vanB* genes are those predominantly encountered in vancomycin-resistant enterococci (VRE). They are transferable and can spread from organism to organism and are the only ones that are clinically relevant. In contrast, *vanC* genes are not transferable, have been associated less commonly with serious infections, and have not been associated with outbreaks [8]. Early screening of patients for VRE carriage to identify those patients that require isolation can be part of an effective infection control program for VRE [9, 10].

In most laboratories conventional culture and identification method are considered the gold standard in screening for VRE and MRSA colonization. Since results need a turnaround time (TAT) of 2–4 days which is not compatible to being proactive in terms of infection prevention and control, a test that has a faster TAT and is comparable to culture in efficiency and cost-effectiveness is needed [11–13]. Recently several rapid diagnostic tests have been introduced that would be very beneficial in decreasing the time to detection (several hours), therefore reducing the risk of nosocomial transmission and infections especially in high risk patients [13–18].

The BD GeneOhm MRSA Assay is a multiplex quantitative real-time PCR assay and is a US Food and Drug administration (FDA) cleared qualitative *in vitro* test for direct detection of nasal colonization by MRSA [1]. Its use on specimens from alternative sites has not been FDA approved. The use of this assay in clinical setting requires confirmation especially on swabs from sites other than the nose [19]. It detects the SCC*mec* cassette (carrying the *mecA* gene) and an *S. aureus* specific sequence located within the *orfX* gene, allowing discrimination between MRSA and methicillin-resistant coagulase-negative staphylococci [1]. 

The BD GeneOhm VanR Assay is a CE-mark *in vitro* test for the rapid detection of vancomycin-resistant (*vanA* and *vanB*) genes directly from perianal and/or rectal swabs. The BD GeneOhm VanR Assay can be used as an aid to identify, prevent, and control vancomycin-resistant colonization in healthcare settings [13]. It is not intended to diagnose VRE infection nor to guide or monitor treatment for VRE. Concomitant cultures are necessary to recover organisms for confirmatory identification [20].

King Fahad Specialist Hospital Dammam (600 bed total capacity) has oncology centre and is establishing bone marrow and solid organ transplant programs and does have guidelines for hospital wide screening of new hospital admission for MRSA and VRE. The development of reliable and rapid method for the identification of patients colonized with MRSA and VRE is central to the containment of this agent within the hospital environment. To this end, we investigated the performance of the BD GeneOhm Assays as an alternative to culture which requires 2-3 days before implementing it as a rapid screening method in the hospital. 

## 2. Materials and Methods

Patients and specimens were screened for MRSA and VRE. In the present study, 2267 duplicate swabs were collected (728 nasal and 1539 groin swabs) between January 2008 and June 2009 from all new hospital admissions. In addition, 300 duplicate rectal swabs were collected consecutively between January 2009 and June 2009 from the all patients admitted to the intensive care unit. Nasal and groin swabs were analyzed for the presence of MRSA by conventional culture method and BD GeneOhm MRSA Assay. Rectal swabs were analyzed for the presence of VRE by culture and BD GeneOhm VanR Assay. The duplicate nasal, groin, and rectal swabs from each patient were obtained by nursing staff using BBL CultureSwab Liquid Stuart single swab (Becton Dickinson). For each assay, the two swabs were randomly separated in the laboratory. One swab was used for direct and enrichment broth culture, while PCR was performed with the other swab. If processing of the swabs was not possible on the same day, swabs were stored overnight at 4°C. The BD GeneOhm MRSA Assay is FDA approved for the direct detection of nasal colonization by MRSA only. However, we used it for both nasal and groin swabs. 

### 2.1. Conventional Culture Identification and Susceptibility Testing for MRSA

Collected swabs were inoculated onto blood agar plate (BA), mannitol salt agar (MSA), Mueller hinton with 4% NaCl and oxacillin (MOX) agar, Columbia polymyxin nalidixic acid and bacitracin agar (PNBA) and then inoculated into salt broth. Plates were incubated at 37°C for 24 h. Suspected colonies were identified by conventional laboratory methods, including Gram stain, Catalase test, and coagulase production by the tube method [21]. Coagulase positive strains were inoculated on oxacillin screen plate and tested for susceptibility to cefoxitin disc and oxacillin *E*-test strips. Strains of *Staphylococcus aureus isolated* directly from the agar and/or broth cultures that give a zone of ≤21 mm with cefoxitin or an MIC of ≥4 with the oxacillin *E*-test were reported as MRSA according to Clinical and Laboratory Standards Institute (CLSI) guidelines [22].

### 2.2. BD GeneOhm MRSA Assay

The BD GeneOhm MRSA Assay was performed as recommended by the manufacturer. The tip of the swab was broken off into an MRSA sample buffer tube containing a Tris-EDTA sample preparation buffer, provided by the manufacturer. After the sample buffer tube was vortexed for 1 min, 50 *μ*L of the solution was transferred into a lysis tube. The lysis tube was vortexed for 5 minutes at high speed and received a quick spin in the centrifuge to bring the contents to the bottom of the tube before incubation at 95°C in a dry heating block for 2 minutes. The sample was kept at 2 to 8°C until PCR testing. The PCR processing was performed as recommended by the manufacturer using the Smart Cycler II instrument (Cepheid, Sunnyvale, Calif, USA). Positive and negative controls were included in each run. The assay results were interpreted as follows: “NEG,” no MRSA DNA was detected; “POS,” MRSA DNA was detected; “unresolved,” the IC was inhibited or there was reagent failure; “not determined,” there was an I-CORE module malfunction.

#### 2.2.1. Quality Control

In addition to the previously mentioned PCR control for each run, a reference MRSA strain (ATCC 4300) and a reference MSSA strain (ATCC 29213) were included in each assay and on day of use with MOX agar.

### 2.3. Conventional Culture Identification and Susceptibility Testing for VRE 

Rectal swabs for culture were first inoculated onto Columbia PNBA and then into salt broth. Plates were incubated at 35°C in ambient air and examined for growth at 24 and 48 hours. Broth cultures were incubated at 35°C. Any suspected colonies were identified by conventional laboratory methods, including Gram stain, Catalase test, bile esculin agar (BEA) test, and BVS (Vancomycin screening agar that incorporates the use of 6 ug/mL of vancomycin in brain-heart infusion (BHI) agar) [21]. Black colonies (esculin-positive) were then subcultured onto a blood agar plate for purity. Following 24 hours of incubation, a definite spot of growth or greater than one colony present at the site of inoculation on the BVS agar indicates that the *Enterococci *may be a VRE [23]. Identification (*E. faecalis/E. faecium*) was confirmed by performing GP on the vitek 2 system (bioMerieux; GP colorimetric identification card). Susceptibility testing was performed on confirmed enterococcal isolates using vancomycin (0.016 to 256 *μ*g/mL) and teicoplanin (0.016 to 256 *μ*g/mL) *E*-test strips. The determination of the MICs and the interpretation of vancomycin resistance (MIC ≥ 32 *μ*g/mL) were done according to Clinical and Laboratory Standards Institute (CLSI) guidelines [21]. For the interpretation of the teicoplanin results, combination of the intermediate and resistant MICs was done as previously published for the assignment of isolates as having *van*A (MIC ≥ 16 *μ*g/mL) or *van*B (MIC < 16 *μ*g/mL) [13]. 

#### 2.3.1. BD GeneOhm VanR Assay

For BD GeneOhm VanR Assay, the assay was performed by following the manufacturer protocol and as described elsewhere [13]. The tip of the swab was broken off into a VanR sample buffer tube containing a Tris-EDTA sample preparation buffer, provided by the manufacturer. After the sample buffer tube was vortexed for 1 min, 50 *μ*L of the solution was transferred into a lysis tube. The lysis tube was vortexed for 5 minutes at high speed and received a quick spin in the centrifuge to bring the contents to the bottom of the tube before incubation at 95°C in a dry heating block for 2 minutes. The sample was kept at 2 to 8°C until PCR testing. The PCR processing was performed as recommended by the manufacturer using the Smart Cycler II instrument (Cepheid, Sunnyvale, Calif, USA). Positive and negative controls were included in each run. The assay results were interpreted as follows: “negative,” no *vanA *or *vanB *DNA was detected; “positive,” *vanA *and *vanB *DNA was detected; “Presumptive POS,” *vanB *DNA was detected; “POS,” *vanA *was detected; “unresolved,” the IC was inhibited or there was reagent failure; “invalid assay run,” the PCR control (positive or negative) failed; “not determined,” there was an I-CORE module malfunction.

#### 2.3.2. Quality Control

In addition to the previously mentioned PCR control for each run, a reference VRE strain (*E. faecalis* ATCC 51299) and a reference *E. faecalis* Vancomycin susceptible strain (ATCC 29212) were included in each assay and on day of use with the BVS agar.

### 2.4. Resolution of Discordant Results

If any of the BD GeneOhm for MRSA and VRE was unresolved, that generally would indicate some amount of PCR inhibition. The assay was repeated once using the frozen lysate as recommended in the kit directions. Unresolved specimens after repetition were excluded from final analysis. For the only sample in which the BD GeneOhm was negative for MRSA but the culture was MRSA positive, the original frozen lysate and the corresponding isolate were tested again by the BD GeneOhm MRSA Assay. BD GeneOhm positive samples, which were culture negative, were examined again by inoculating 300 *μ*L of cell suspension from the sample buffer including the swab into a salt enrichment broth. This was followed by incubation for 18–24 hours in air at 35°C and the subculture onto appropriate culture media.

### 2.5. Statistical Analysis

Descriptive statistical performance characteristics were calculated for the BD GeneOhm MRSA and VanR Assays relative to gold standard direct culture and broth enrichment culture results. The sensitivity, specificity, positive predictive value (PPV), and negative predictive value (NPV) were calculated according to standard formulas. The commercial statistical software package used was SPSS 11.0 (SPSS, Inc., Chicago, ILL, USA) and was used for statistical evaluation of results.

## 3. Results

For detection of MRSA, a total of 2283 swabs were collected and tested as described above. Thirty-two (32) specimens were inhibited in the PCR assay with an initial unresolved rate being 1.4% of which 16 (0.7%) were resolved following freeze thaw of the lysate and repeat of PCR testing. The remaining 16 unresolved samples were excluded in the data analysis resulting in 2267 total swabs. The 2267 swabs (728 nasal and 1539 groin swabs) were tested. Of the 728 nasal swabs, 35 swabs (4.8%) revealed MRSA by culture. The 1539 groin swabs revealed MRSA in 32 swabs (2.1%) by culture. In the BD GeneOhm MRSA Assay, 39 nasal swabs (5.4%) were positive and 52 groin swabs (3.4%) were positive. Tables 1 and 2 show the distribution of positive and negative samples by both methods. One MRSA nasal isolate was detected by culture and was negative by PCR (false negative). Four nasal (0.5%) and 20 groin swabs (1.3%) were positive with the BD GeneOhm MRSA Assay and did not reveal MRSA in culture (false positive).

The BD GeneOhm MRSA Assay showed sensitivity (the proportion of actual MRSA positives which are correctly identified as such), specificity (the proportion of actual MRSA negatives which are correctly identified as such), PPV (the proportion of positive MRSA with PCR who are correctly diagnosed), and NPV (the proportion of negative MRSA with PCR who are correctly diagnosed) of 97.2%, 99.4%, 89.7% and 99.9%, respectively for direct detection from nasal swabs. For direct detection from groin samples, it was 100%, 98.7%, 61.5%, and 100%, respectively.  Table 3 shows sensitivity, specificity, PPV, and NPV of the BD GeneOhm MRSA Assay using nasal and groin swabs.

The 300 rectal swabs revealed VRE in 8 samples by culture, and 292 samples were negative. The 8 strains were *E. faecium* and all of them were resistant to vancomycin and sensitive to teicoplanin (phenotype B). The overall prevalence of VRE by culture was 2.7%. The BD GeneOhm VanR Assay detected *vanB* gene in 34 samples (11.3%). *vanA* gene was not detected in any sample. Twenty-six rectal swabs (8.7%) revealed *vanB* gene by BD GeneOhm VanR Assay but did not grow enterococci in culture (false +ve). The distribution of positive and negative samples by both methods is shown in Table 4. Compared to conventional culture, the BD GeneOhm VanR Assay showed a sensitivity (the proportion of actual VRE positives which are correctly identified as such), specificity (the proportion of actual VRE negatives which are correctly identified as such), PPV (the proportion of positive VRE with PCR who are correctly diagnosed), and NPV (the proportion of negative VRE with PCR who are correctly diagnosed) of 100%, 91.1%, 23.5%, and 100%, respectively. 

## 4. Discussion

Active surveillance culture from patients for carriage of MRSA and VRE facilitates early contact isolation, thus preventing spread in hospital and reducing costs [1]. King Fahad specialist hospital-Dammam has guidelines for hospital wide screening of new hospital admissions for MRSA and VRE. The time to result with conventional culture is 2–4 days. A rapid, accurate tool that identify carriers is a key component of any infection control program to reduce transmission. In this study, we evaluated the detection performance of the BD GeneOhm VanR Assay, a rapid real-time PCR test that detects the presence of *vanA* and/or *vanB*, and the BD GeneOhm MRSA Assay which detects SCCmec cassette and *S. aureus* specific sequence located within the *orfX* gene, allowing discrimination between MRSA and methicillin-resistant coagulase-negative staphylococci. BD GeneOhm for both MRSA and VanR Assays offer rapid identification of MRSA and VRE colonized patients in as little as 2 hours [13].

The BD GeneOhm MRSA Assay was compared to culture. The BD GeneOhm MRSA Assay showed a high specificity with nasal and groin samples (99.4% and 98.7%, resp.). The sensitivity was also higher in our group of patients (97.2% and 100%) compared with that of Lucke et al. [24] who reported a lower sensitivity (84.3%). This may be attributed to the type of specimens that was collected from different clinical conditions and from different sites. Screening for MRSA from groin swabs is considered a modified FDA-cleared test. Therefore, we examined 1539 specimens to come across a minimum of 50 specimens that contain the target analyte and a minimum of 100 specimens that lack the target analyte [25]. 

There is a general consensus for multiple body site screening to achieve optimal detection of MRSA. Considering pooling of the specimen may reduce the cost of the assay; Bishop et al. [26] showed sensitivities and specificities for pooled nose-groin specimen comparable to those processed separately with the BD GeneOhm MRSA Assay.

In our group, the PPV was 89.7% with the nasal swabs and it was diminished to 61.5% with the groin swabs. Recent studies [19, 27] reported PPVs ranging from 63% in a community setting to 94% in hospital setting. The high NPV in this study (nasal 99.9% and groin 100%) and those reported by others [28, 29] suggest that this assay provides a rapid method for the identification of persons who are not colonized with MRSA and in that context is likely to be useful for epidemiologic or surveillance activity in a hospital environment. The 24 swabs that detected MRSA by the BD GeneOhm MRSA Assay and were not recovered in culture may belong to patients receiving antibiotics [30]. The BD GeneOhm MRSA Assay result may sometimes be unresolved and may require retesting that can lead to a delay in obtaining final results. This problem was met with 32 of our samples and half of them were resolved after repeat testing of the corresponding frozen lysates for specimen and controls. The frozen-thaw cycles have been shown to reduce PCR inhibitors substances in the specimen lysate [29, 30]. One of our MRSA isolates from the nose was not detected by the BD GeneOhm MRSA Assay giving false negative result. This was also reported in a study done by Bartels et al. in Copenhagen [31], as more than one-third of their MRSA isolates were not detected. They recommended that the BD GeneOhm MRSA Assay be evaluated against the local MRSA diversity before being established as a standard assay, and due to the constant evolution of SCCmec cassettes, a continuous global surveillance is advisable in order to update the assay [31]. In the present study, the BD GeneOhm MRSA Assay had a high sensitivity (97% and 100% for nasal and groin swabs, resp.) and thus, can be regarded as reliable in our hospital.

In conclusion, the BD GeneOhm MRSA Assay represents a reliable screening test when applied to nasal and groin specimens. The true strength of this assay is its exceptionally high NPV (98.9%) making the test an ideal tool for rapid exclusion of MRSA carriers in hospitals. As a consequence, this would dramatically shorten the patient isolation time.

In this study, the BD GeneOhm VanR Assay detected *van*B gene in 34 samples, eight of them revealed *E. faecium* that were phenotypically *vanB*. The BD GeneOhm VanR Assay has specificity 91.1% with a positive predictive value of 23.5%. This poor positive predictive value with the assay was also reported by other workers [32]. This was explained by the high prevalence of *van*B genes not associated with VRE from human rectal swabs. Graham et al. [33] demonstrated high rates of nonenterococcal *van*B carriage in hemodialysis patients (45%), community adults (63%), and children (27%). This is attributed to the presence of gut anaerobes carrying the *van*B containing transposons Tn 5382 and Tn 1549. Hence, a positive *van*B PCR is poorly predictive and requires culture to differentiate VRE positive patients from VRE negative patients (i.e., PCR false positive) and hence, the *van*B gene is the only one detected in our group of patients. Relying on a positive *van*B result in our hospital would result in unnecessary utilization of hospital resources and infection control prevention measures for patients who are not harboring VRE. In this study, the BD GeneOhm vanR negative predictive value was 100%. We need to weigh the convenience of rapid negative results with the requirement for additional testing. In conclusion, the BD GeneOhm vanR Assay may be used as a rapid screening method for *vanA* and *vanB* carriers, given the high sensitivity and negative predictive value. However, the high number of false positive for *vanB* will necessitate culture confirmation of these results.

## Figures and Tables

**Table 1:** Comparison of BD GeneOhm MRSA and culture in the detection of MRSA with nasal swabs.

Nasal swabs		Culture method		
		POS	Negative	Total
BD GeneOhm MRSA PCR Assay	POS	35	4	39
	Negative	1	688	689
	Total	36	692	728

**Table 2:** Comparison of BD GeneOhm MRSA Assay and culture in the detection of MRSA with groin swabs

Groin swabs		Culture method		
		POS	Negative	Total
BD GeneOhm MRSA PCR Assay	POS	32	20	52
	Negative	0	1487	1487
	Total	32	1507	1539

**Table 3:** Sensitivity, specificity, positive predictive value (PPV), and negative predictive value (NPV) of the BD GeneOhm Assay MRSA real-time PCR using nasal and groin swabs.

Value % (95% CI)	Type of sample	
	Nasal swabs (728)	Groin swabs (1539)
Sensitivity^1^	97.2% (0.83–0.99)	100% (0.86–1)
Specificity^2^	99.4% (0.98–0.99)	98.7% (0.97–0.99)
PPV^3^	89.7% (0.74–0.96)	61.5% (0.47–0.74)
NPV^4^	99.9% (0.99–0.99)	100% (0.99–1)

^1^The proportion of actual positives by PCR which are correctly identified as such.

^2^The proportion of actual negatives by PCR which are correctly identified as such.

^3^The proportion of positive test results with PCR who are correctly diagnosed.

^4^The proportion of negative test results with PCR who are correctly diagnosed.

**Table 4:** Comparison of BD GeneOhm VanR Assay and culture in the detection of VRE with rectal swabs.

Rectal swabs		Culture method		
		POS	Negative	Total
BD GeneOhm VanR PCR assay	POS	8	26	34
	Negative	0	266	266
	Total	8	292	300

(Sensitivity: 100%; specificity: 91.1%; positive predictive value (PPV): 23.5%; negative predictive value (NPV): 100%).
